# Hyperbaric oxygen‐induced long non‐coding RNA MALAT1 exosomes suppress MicroRNA‐92a expression in a rat model of acute myocardial infarction

**DOI:** 10.1111/jcmm.15889

**Published:** 2020-09-16

**Authors:** Kou‐Gi Shyu, Bao‐Wei Wang, Wei‐Jen Fang, Chun‐Ming Pan, Chiu‐Mei Lin

**Affiliations:** ^1^ Division of Cardiology Shin Kong Wu Ho‐Su Memorial Hospital Taipei Taiwan; ^2^ Department of Research Shin Kong Wu Ho‐Su Memorial Hospital Taipei Taiwan; ^3^ Department of Emergency Medicine Shin Kong Wu Ho‐Su Memorial Hospital Taipei Taiwan; ^4^ School of Medicine Fu‐Jen Catholic University New Taipei City Taiwan

**Keywords:** exosome, hyperbaric oxygen, MALAT1, myocardial infarction

## Abstract

Hyperbaric oxygen (HBO) improves angiogenesis. The effect of HBO on metastasis‐associated lung adenocarcinoma transcript 1 (MALAT1), a pro‐angiogenic long non‐coding RNA, in cardiac myocyte‐derived exosomes and acute myocardial infarction (AMI) is unknown. We aimed to investigate whether MALAT1 is altered in cardiac myocyte‐derived exosomes in response to HBO as well as the molecular regulatory mechanisms of MALAT1 in cardiac myocytes treated with HBO. Cardiac myocytes were cultured, and HBO was applied at 2.5 atmosphere absolute in a hyperbaric chamber. Exosomes were extracted from the culture media. A rat model of AMI generated by the ligation of the left anterior descending artery was used. HBO significantly increased MALAT1 expression in cardiac myocytes and HBO‐induced MALAT1 and exosomes attenuated miR‐92a expression after myocardial infarction. Expression of krüppel‐like factor 2 (KLF2) and CD31 was significantly decreased after infarction and HBO‐induced exosomes significantly reversed the expression. Silencing of MALAT1 using MALAT1‐locked nucleic acid GapmeR significantly attenuated KLF2 and CD31 protein expression after infarction induced by HBO‐induced exosomes. HBO‐induced exosomes also decreased infarct size significantly. HBO‐induced exosomes from cardiac myocytes up‐regulate MALAT1 to suppress miR‐92a expression and counteract the inhibitory effect of miR‐92a on KLF2 and CD31 expression in left ventricular myocardium after myocardial infarction to enhance neovascularization.

## 1 INTRODUCTION

Acute myocardial infarction (AMI) and the ensuing heart failure are major causes of morbidity and mortality worldwide. The loss of blood flow to the myocardium as a result of occlusion of the coronary artery results in the death of cardiac myocytes and myocardial remodelling. Cardiac remodelling after AMI reduces left ventricular contractility and eventually results in heart failure.^1^ Improvement of myocardial ischaemia by enhancement of neovascularization following AMI may improve cardiac function and reduce heart failure events. The enhancement of myocardial neovascularization by therapeutic angiogenesis represents an important therapeutic strategy in the management of post‐myocardial infarction (MI).

Hyperbaric oxygen (HBO) therapy significantly increases the oxygen content in hypoperfused tissues, and elevation of oxygen content in the hypoxic tissues promotes the ischaemic repair process.^2^, ^3^ One of the mechanisms through which HBO therapy improves wound healing is through the enhancement of neovascularization in the ischaemic tissue.^4^ HBO was shown to induce cardioprotection through anti‐apoptotic effect and decreased infarct size in a rat model of MI.^5^, ^6^ Several small clinical trials also demonstrated the beneficial effect of HBO in patients with acute coronary syndrome by reducing the infarct size, and the risk of major cardiovascular events and death.^7^

Exosomes are 40‐90 nm‐sized extracellular vesicles of endosomal origin that play a crucial role in intercellular processes.^8^, ^9^ Exosomes are an emerging and promising class of diagnostic markers for cell‐free therapeutic application in cardiovascular diseases.^9^, ^10^ Giricz et al^11^ reported that exosomes released from the rat heart following ischaemic pre‐conditioning are important and responsible for the transmission of remote conditioning signals for cardioprotection. Sahoo et al^12^ demonstrated that both ischaemic and healthy human and mouse cardiac myocytes may release exosome‐like vesicles in vivo to repair MI injury. These results indicate that exosome‐mediated communication mechanisms may play a significant role in cardiovascular repair and regeneration. This new therapeutic strategy may lead to the discovery of novel mechanisms for the treatment of cardiovascular diseases. The endothelial‐enriched long non‐coding RNA (lncRNA), metastasis‐associated lung adenocarcinoma transcript 1 (MALAT1), is considered to be a pro‐angiogenic lncRNA because the loss of MALAT1 impairs neovascularization in vitro and in vivo.^13^ MALAT1 plays a role in the promotion of angiogenesis by mesenchymal stem cells in thyroid tumours.^14^, ^15^ The effect of HBO on MALAT1 expression in MI remains unknown. It is also not known whether HBO‐induced exosomes have a therapeutic potential post‐MI. In this study, we used HBO‐induced MALAT1 exosomes from cardiac myocytes to treat MI in an animal study.

## 2 METHODS

### 2.1 Rat model of acute myocardial infarction (AMI)

A rat model of AMI with left anterior descending (LAD) coronary artery ligation was used as previously described.^16^ Wistar male rats weighing 280‐330 g were used. Following the induction of anaesthesia with 2% isoflurane and confirmation of a fully anaesthetized state (no response to toe pinching), tracheotomy was performed, and the animal was ventilated on a Harvard rodent respirator. The heart was then rapidly exteriorized, and a 6‐0 silk suture was tightened around the proximal LAD coronary artery (before the first branch of diagonal artery). Sham‐operated control animals were prepared in a similar manner without LAD ligation. Following the surgical procedure, the wound and tracheotomy were closed to restore spontaneous respiration. For the AMI study, rats were randomly divided into nine groups (a) sham‐operated, (b) AMI alone, (c) AMI and treatment with HBO, (d) AMI and treatment with HBO‐induced exosomes, (e) AMI and treatment with MALAT1‐locked nucleic acid (LNA) GapmeR (Cat. no. 339511 LG00157651‐DDA, Qiagen), (f) AMI and treatment with MALAT1‐scrambled LNA GapmeR (Cat. no. 339511 LG00199141‐DDA, Qiagen), (g) AMI and treatment with antagomir‐92a, (h) treatment with miR‐92a in sham control and (i) treatment with mutant‐miR‐92a in sham control. After 60 minutes, the viable myocardium bordering the LV infarct zone was injected at three different sites with a total of 70 μg of MALAT1‐containing exosomes (150 μL). During the HBO sessions, the rats were kept in separate restrainers in the hyperbaric chamber. Oxygen flow (100%) through the chamber was set at 2.5 L/min to maintain the room temperature (RT). Rats in the AMI + HBO group received HBO treatment once a day for 60 minutes at 2.5 atmosphere absolute (ATA) for various time periods. The oxygen tension mentioned above were chosen based on the current human treatment protocols.^17^ Rats in the sham group were treated with normobaric air at 1.0 ATA. At the end of experiment, the rats were killed by decapitation under anaesthesia with an overdose of isoflurane, and the heart was quickly removed and stored in liquid nitrogen. Left ventricular tissue was obtained for Western blot analysis and immunofluorescent staining. The infarct size was measured using the triphenyl‐tetrazolium chloride method. Computerized morphometry (NIS Elements, Nikon) was used to calculate the infarct size (as the ratio of infarct size and total left ventricular area). All animal procedures were performed in accordance with institutional guidelines and conformed to the Guide for the Care and Use of Laboratory Animals published by the United States National Institutes of Health.

### 2.2 Haemodynamic monitoring of rats

Haemodynamic monitoring of the rats was performed with polyethylene catheters for measurement through a Grass model tachograph pre‐amplifier as previously described.^16^

### 2.3 Assessment of cardiac function

Cardiac function of the rats was evaluated non‐invasively using echocardiography performed with an Acuson Sequoia 512 machine using a 15‐MHz probe on the day of killing, and on days 7 and 14 following the surgery as described previously.^16^ The sonographer was blinded to the randomization of the rats.

### 2.4 Reverse transcription and real‐time quantitative polymerase chain reaction

We used high‐capacity cDNA reverse transcription kits (Applied Biosystems, Thermo Fisher Scientific) to quantify MALAT1‐exosomes, miR‐92a and krüppel‐like factor 2 (KLF2) mRNA transcripts. The RNA eluate (12 μL out of the 14‐μL) was subjected to reverse transcription with random hexamers. The primers used were as follows: MALAT1: 5′‐TGCATTGTCTCTGTAGGTGTCTCTCT‐3′ (forward) and 5′‐TGGGCCCTCGAATAGATGTC‐3′ (reverse); krüppel‐like factor 2 (KLF2): 5′‐ACCAACTGCGGCAAGACCTA‐3′ (forward) and 5′‐CCGTGTGCTTGCGGTAGTG‐3′ (reverse); α‐tubulin: 5′‐GTGG TCCCCAAAGATG‐3′ (forward) and 5′‐CCCTCCACCGAATCAA‐3′ (reverse). Reverse transcription was performed as previously described.^18^ MiR‐92a were purchased from Thermo Fisher Scientific (Assay ID 000431).

### 2.5 Western blot analysis

Western blotting was performed as previously described.^19^ The proteins of interest were identified through incubation with specific antibodies, as indicated (1:1000 dilution), for 1 hour at RT. Equal protein loading of the samples was further verified by staining with mouse anti‐tubulin monoclonal antibody (Santa Cruz Biotechnology Inc). All Western blot signals were visualized using chemiluminescence and quantified using densitometry.

### 2.6 Histological and immunofluorescence staining

Tissue samples were harvested and treated overnight in 4% paraformaldehyde and then embedded in paraffin. The tissue were cut into 5‐μm‐thick sections and incubated with the primary antibody at 4°C for 12 hours. The sections were then washed thrice with PBS, incubated with fluorescence‐conjugated secondary antibody in PBS for 1‐2 hours at RT in the dark and then stained with DAPI to visualize the nuclei. The sections were mounted with a coverslip and examined using fluorescence microscopy. Images were taken from at least three random fields for each sample.

### 2.7 Transfection of siRNAs and MALAT1 LNA GapmeR

The LNA GapmeR was transfected into left ventricular myocardium using a low pressure‐accelerated gene gun (Bioware Technologies) following the protocol from the manufacturer. In brief, 1 µmol/L of LNA GapmeR was suspended in 5 mL of PBS and placed in the loading hole near the nozzle. Pushing the trigger of the gene gun released the RNA‐containing solution, which was directly propelled by helium at a pressure of 15 psi into left ventricular myocardium of the MI rat. Scrambled siRNA or LNA GapmeR was transfected as negative controls.

### 2.8 Culture of rat cardiac myocytes

Rat cardiac myocytes were obtained from ScienCell Research Laboratories (Cat. No. R6200; ScienCell). The cardiac myocytes were isolated from postnatal day 2 rat heart, cryopreserved at P0 and characterized by immunofluorescence using antibodies specific to smooth muscle actin, sarcomeric alpha actinin and tropomyosin. The cardiac myocytes were plated in a culture dish and cultured in cardiac myocyte medium (Cat. No. 6201; ScienCell) according to the manufacturer's instructions. After 3 days in culture, the cells were transferred to serum‐free Dulbecco's modified Eagle's medium (DMEM, Cat. No. 31600‐034; Invitrogen Corporation) and used for the experiments.

### 2.9 Hypoxic stimulation

A humidified temperature‐controlled incubator, Proox model 110 (BioSpherix) was used as the hypoxia chamber. For hypoxic conditions, the concentration of oxygen was reduced to 2.5% by replacement with N2, keeping CO_2_ constant at 5%, and cells were incubated at 37°C for different time periods. Control condition was defined as 95% air and 5% CO_2_. To investigate the molecular regulatory mechanisms, cardiac myocytes were pre‐treated with inhibitors for 30 minutes and then exposed to hypoxia without changing the medium.

### 2.10 Extraction of exosomes from cell culture media

Exosomes were isolated from the cell culture media using Total Exosome Isolation Reagent (Invitrogen) according to the manufacturer's instructions. Briefly, the cell‐free supernatant was centrifuged at 2000 g for 30 minutes to remove the cell debris. The supernatant containing the cell‐free media was transferred to a fresh container and incubated on ice until use. Each sample was combined with 1/2 volume of Total Exosome Isolation Reagent and mixed well by vortexing or pipetting up and down until a homogenous solution was formed. The samples were incubated at 4°C overnight and then centrifuged at 10 000 g for 1 hour at 4°C. The supernatant was aspirated and discarded, and the exosome pellet was resuspended in 1× PBS, and then stored at 4°C for short‐term (1‐7 days) or −20°C for long‐term storage. The exosomes were quantitated using ExoQuant™ quantification assay kit (BioVision) according to the manufacturer's instructions.

### 2.11 Assessment of the quality of exosomal‐RNA

The quality of exosomal‐RNA was assessed with an Agilent 2100 Bioanalyzer using an RNA Pico Chip (Agilent Technologies) following the manufacturer's protocol. For visualization and better interpretation, electropherograms and virtual gel images were generated. All chip analyses were performed in duplicate.

### 2.12 Luciferase activity assay

A 500‐bp DNA fragment in the 3′‐UTR of rat KLF2 (Chromosome 16:19 223 087‐19 225 037 reverse strand; http://www.ensembl.org/index.html) was artificially synthesized. The amplified product was ligated into pmirNanoGLO luciferase vectors (Promega) with Sac I and Xba I restriction enzymes. The KLF2 3′‐UTR contains an evolutionarily conserved miR‐92a‐binding site (from 205 to 225 bp). For generation of the mutant, the sequence (AATGGTGCAATA) of the binding site was mutated to CATGTGTACCGC and subcloned using the method mentioned above. All the cloned plasmids were verified through DNA sequencing (Seeing Bioscience Co. Ltd.). The plasmids were transfected into cardiac myocytes using a low pressure‐accelerated gene gun (Bioware Technologies) according to the manufacturer's protocol. The test plasmid (2 μg) and the control plasmid (pGL4‐Renilla luciferase, 0.02 μg) were cotransfected with the gene gun in each well, and then, the medium was replaced with normal culture medium. Following 2 hours of exposure to hypoxic condition at 2.5% oxygen, cell extracts were prepared using the Nano‐Glo dual‐luciferase reporter assay system (Promega) and measured for luciferase activity by using a luminometer (Glomax Multi Detection System, Promega).

### 2.13 Statistical analysis

The data are expressed as the mean ± standard deviation (SD). Statistical significance was analysed using analysis of variance (ANOVA; GraphPad Software Inc). The Tukey‐Kramer comparison test was used to conduct pairwise comparisons between multiple groups after ANOVA *P* < .05 was considered statistically significant.

## 3 RESULTS

### 3.1 Effect of HBO and HBO‐induced exosomes on MALAT1, miR‐92a and KLF2 expression

**Figure 1:**
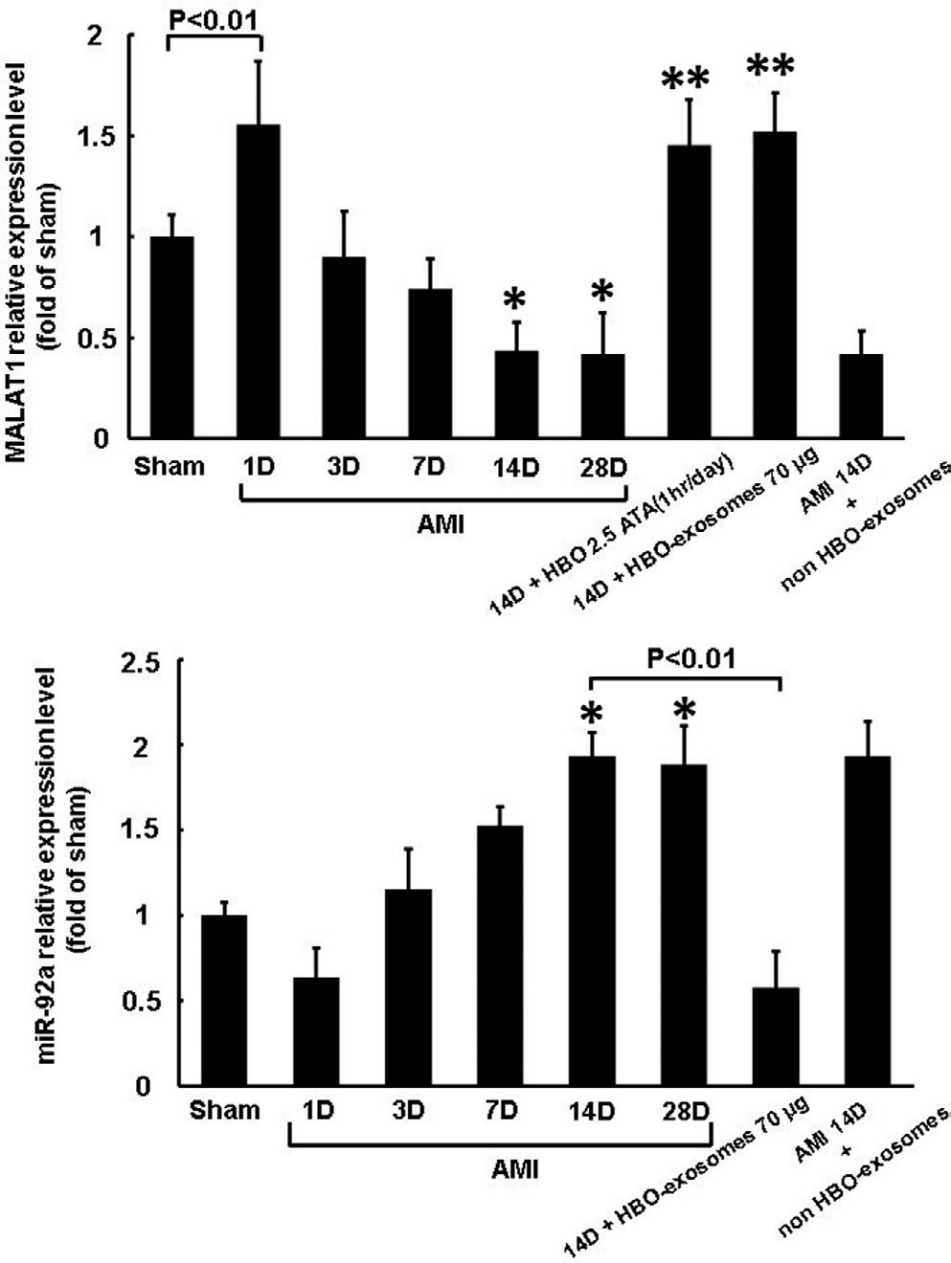
Effect of HBO and HBO‐induced exosomes on MALAT1 and miR‐92a expression in rat left ventricular myocardium in a rat model of acute myocardial infarction. A, Quantitative real‐time PCR analysis of MALAT1 expression at different time points following AMI. *P < .01 vs sham, **P < .001 vs 14 d. B, Quantitative real‐time PCR of miR‐92a expression in different time points following AMI. *P < .01 vs sham (n = 5 per group)

**Figure 2:**
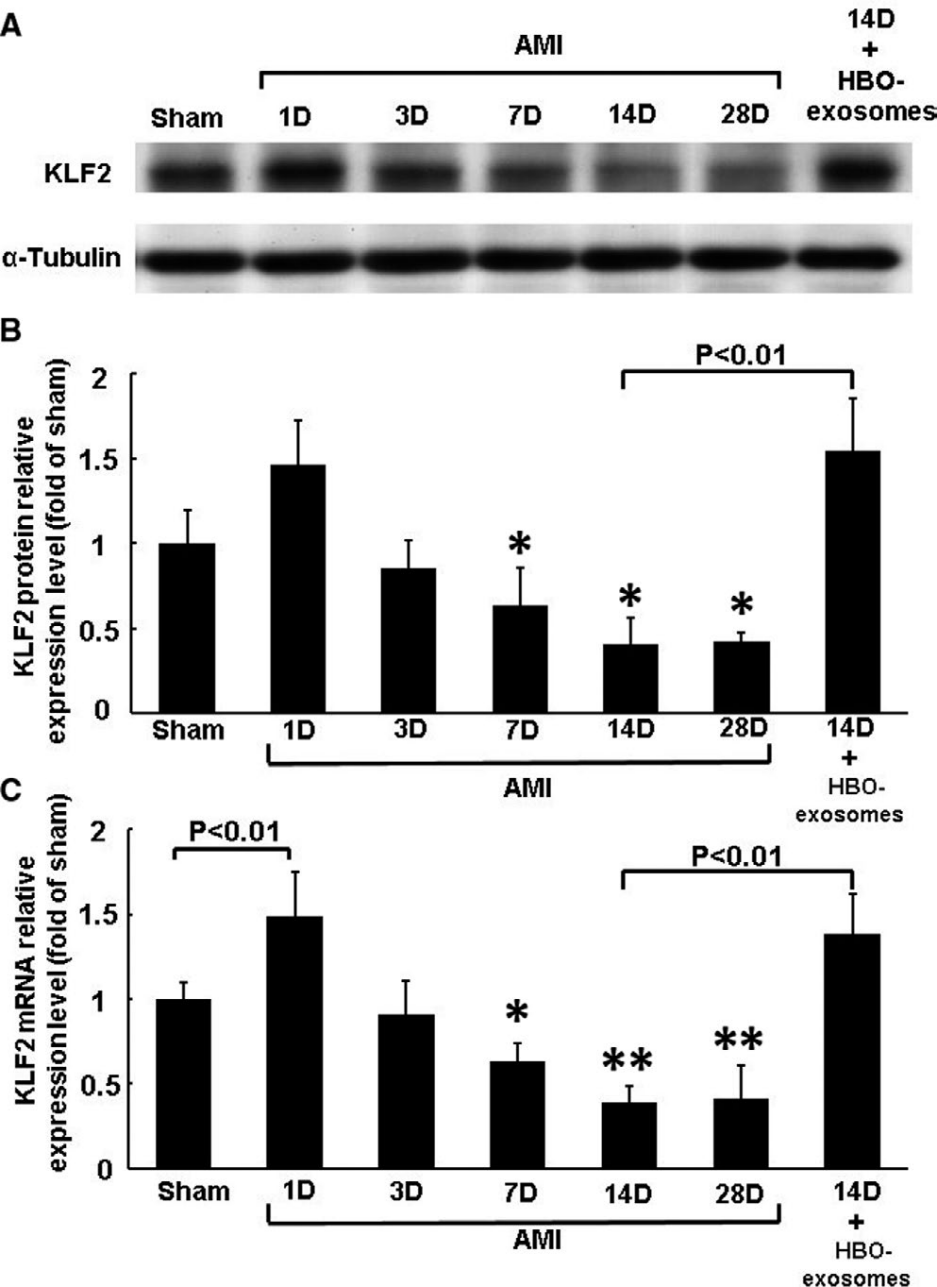
Effect of HBO and HBO‐induced exosomes on KLF2 expression in rat left ventricular myocardium in a rat model of acute myocardial infarction. A, Representative Western blot analysis for KLF2 and α‐tubulin protein expression in rat left ventricular myocardium on different days following AMI. B, Quantitative analysis of KLF2 protein levels. The values of protein expression from left ventricular myocardium after AMI have been normalized to α‐tubulin measurements and then expressed as a ratio of normalized values to protein in the sham group (n = 5 per group). *P < .01 vs sham. C, Quantitative real‐time PCR of KLF2 mRNA expression in rat left ventricular myocardium on different days following AMI. *P < .05 vs sham. **P < .01 vs sham (n = 5 per group)

The expression of MALAT1 in the left ventricular myocardium increased significantly after 1 day and then decreased gradually, and the decrease was significant 14 and 28 days post‐MI (Figure 1A). Treatment with HBO at 2.5 ATA and HBO‐induced exosomes for 14 days post‐MI significantly increased MALAT1 expression compared with AMI alone without any treatment at 14 days (Figure 1A). Treatment with non‐HBO exosomes did not affect MALAT1 expression compared with AMI alone at 14 days. The expression of miR‐92a in the left ventricular myocardium increased significantly 14 and 28 days post‐MI and treatment with HBO‐induced exosomes post‐MI significantly reduced miR‐92a expression compared with AMI alone at 14 days (Figure 1B). Treatment with non‐HBO exosomes did not affect miR‐92a expression compared with AMI alone at 14 days. The expression of KLF2 protein in left ventricular myocardium significantly decreased from 7 to 28 days post‐MI. Treatment with HBO‐induced exosomes post‐MI significantly increased KLF2 protein expression compared with AMI alone at 14 days (Figure 2A,B). KLF2 mRNA expression significantly increased at 1 day and significantly decreased from 7 to 28 days post‐MI and treatment with HBO‐induced exosomes post‐MI significantly reversed the KLF2 expression compared with AMI alone at 14 days (Figure 2C).

### 3.2 MALAT1 and miR‐92a mediate myocardial KLF2 and CD31 expression

**Figure 3:**
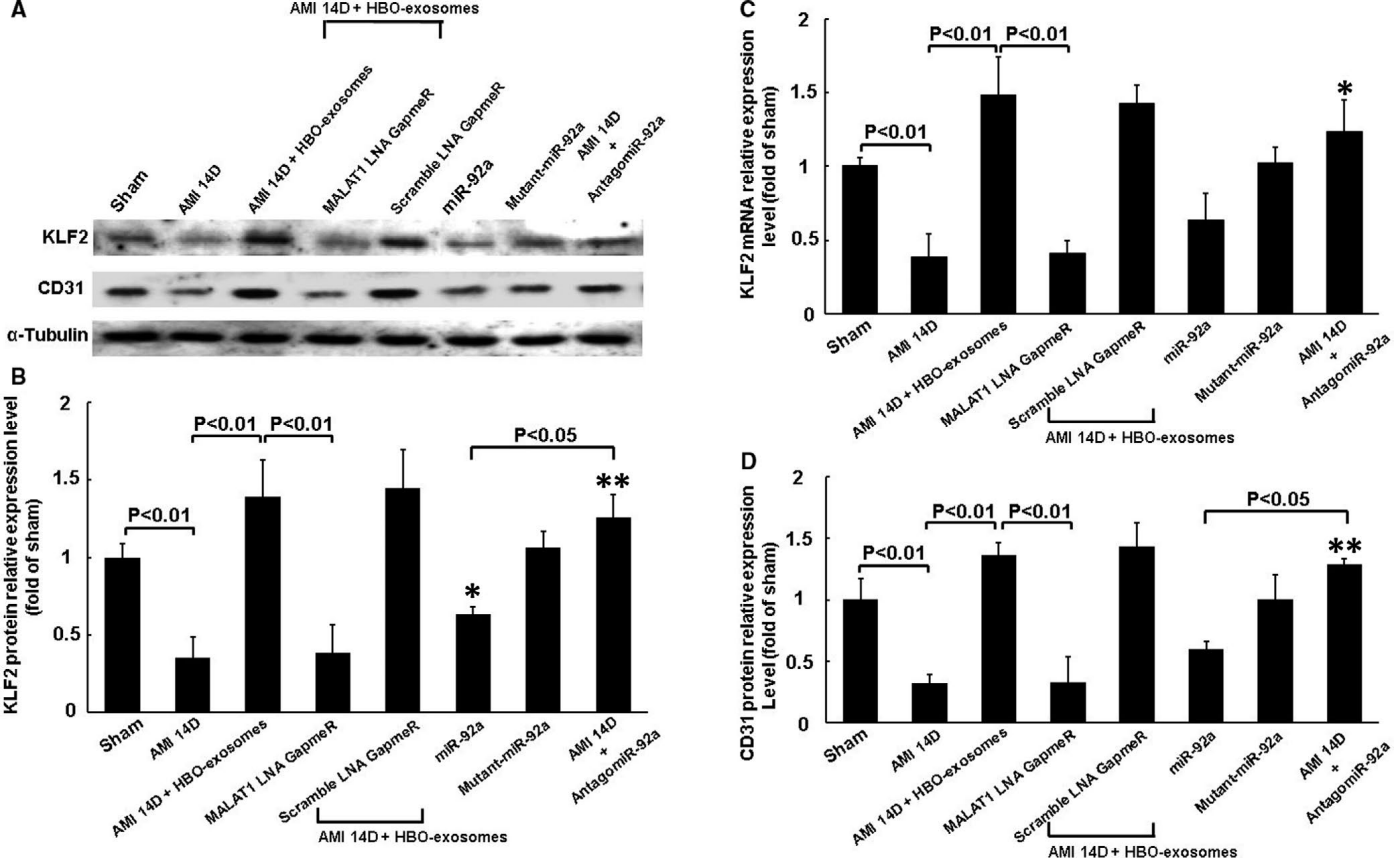
MALAT1 and miR‐92a mediate myocardial KLF2 and CD31 expression in AMI rats. A, Representative Western blot analysis for KLF2, CD31 and α‐tubulin protein expression in the rat left ventricular myocardium. MALAT1 LNA GapmeR and miR‐92a expression vector were transfected into left ventricular myocardium using a low pressure‐accelerated gene gun. B, Quantitative analysis of KLF2 protein levels. The values of protein expression from left ventricular myocardium after AMI were normalized to α‐tubulin measurement and then expressed as a ratio of normalized values to protein in the sham group. *P < .05 vs sham. **P < .01 vs AMI 14 d (n = 5 per group). C, Quantitative real‐time PCR of KLF2 mRNA expression in rat left ventricular myocardium. *P < .01 vs AMI 14 d (n = 5 per group). D, Quantitative analysis of CD31 protein levels. The values of protein expression from left ventricular myocardium after AMI were normalized to α‐tubulin measurement and then expressed as a ratio of normalized values to protein in sham group. *P < .05 vs sham. **P < .01 vs AMI 14 d (n = 5 per group)

**Figure 4:**
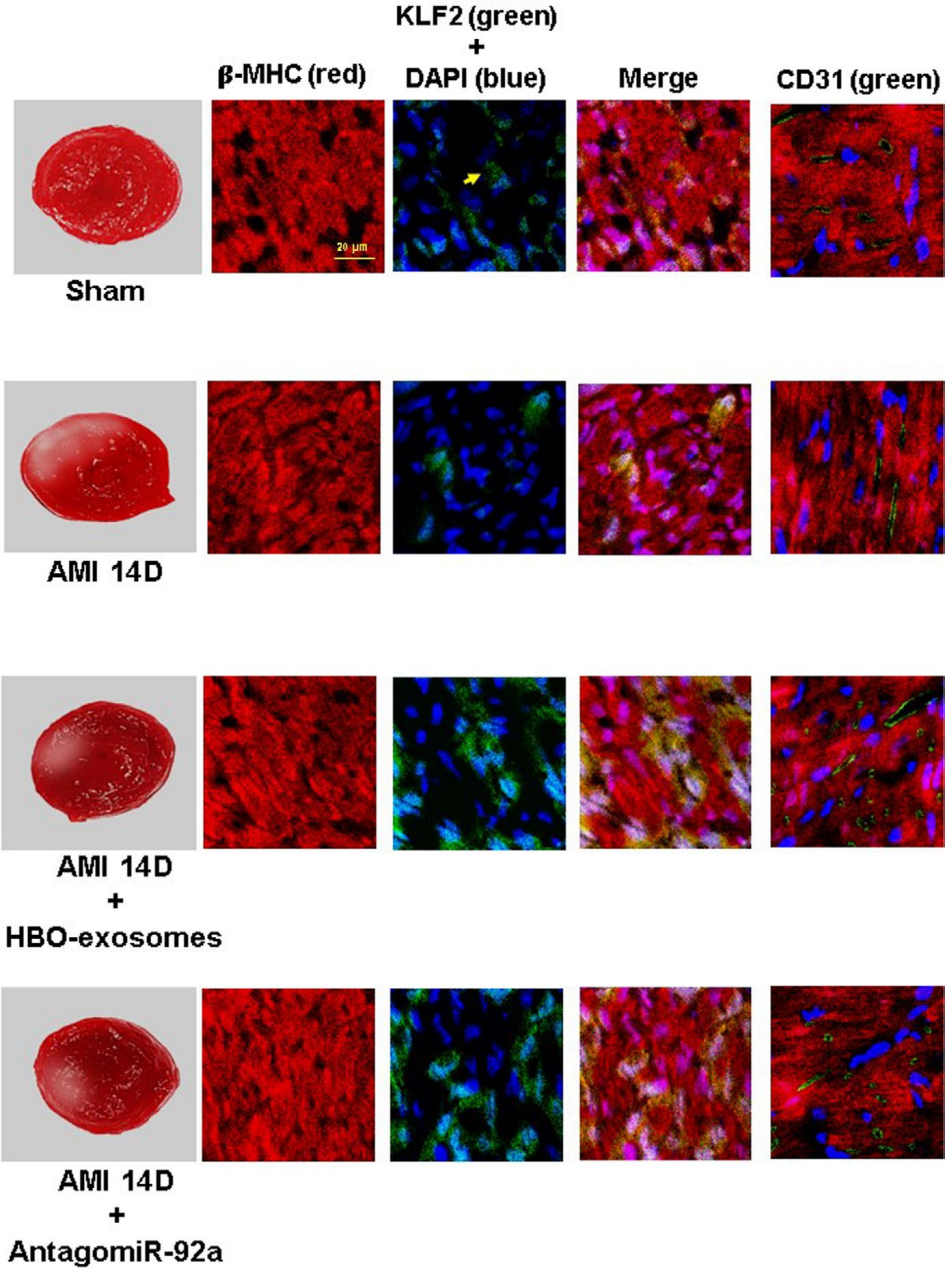
Immunohistochemical staining of left ventricular myocardium after induction of AMI with or without treatment with HBO‐induced exosomes or antagomir‐92a. Immunoreactive signals for KLF2 (yellow colour) and CD31 (green colour) significantly decreased at 14 d post‐AMI. HBO‐induced exosomes and antagomir‐92a significantly increased the immunoreactive signal post‐MI. Representative of four independent experiments

To investigate the effect of MALAT1 and miR‐92a on myocardial KLF2 and CD31 expression, the left ventricular myocardium was transfected with MALAT1 LNA GapmeR and antagomir‐92a overexpression construct. KLF2 and CD31 significantly decreased 14 days post‐MI and HBO‐induced exosomes significantly reversed KLF2 and CD31 expression at 14 days post‐MI (Figure 3). MALAT1 LNA GapmeR significantly attenuated KLF2 and CD31 protein expression at 14 days post‐MI that was enhanced by HBO‐induced exosomes. Scrambled LNA GapmeR of MALAT1 did not attenuate KLF2 and CD31 expression. Overexpression of miR‐92a without MI significantly decreased myocardial KLF2 and CD31 protein expression whereas overexpression of antagomir‐92a in the MI rats at 14 days significantly reversed KLF2 and CD31 expression. Overexpression of mutant‐miR‐92a without MI did not decrease myocardial KLF2 and CD31 protein expression. Silencing of KLF2 by KLF2 siRNA significantly attenuated the CD31 protein expression at 14 days post‐MI that was enhanced by HBO‐induced exosomes (Figure S1). Immunofluorescence staining showed that KLF2 and CD31 signals decreased post‐MI compared with the sham group. Treatment with HBO‐induced exosomes and overexpression of antagomir‐92a increased the KFL2 and CD31 signals at 14 days post‐MI compared with MI alone at day 14 (Figure 4). These results indicate that MALAT1 and miR‐92a mediate myocardial KLF2 and CD31 expression following AMI. Further, we confirmed that MALAT1 from the HBO‐stimulated cardiac myocytes was incorporated into the cardiac myocytes in rat left ventricular myocardium using in situ hybridization (Figure S2).

### 3.3 HBO‐induced exosomes decrease myocardial infarct size

**Figure 5:**
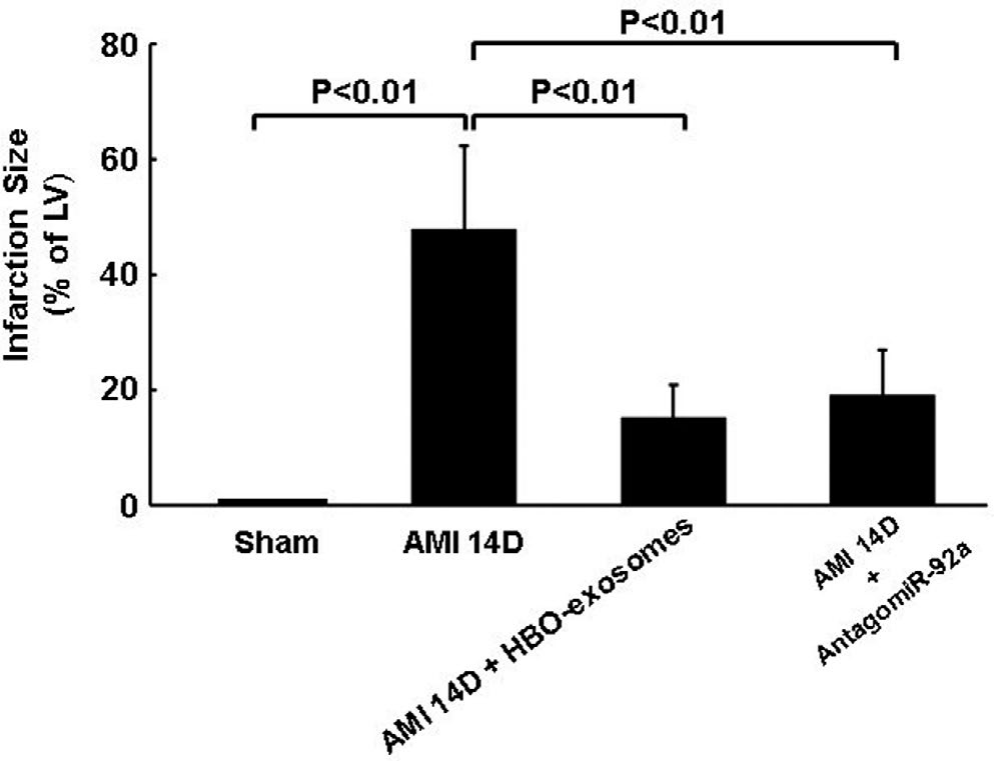
HBO‐induced exosomes and antagomir‐92a significantly decrease myocardial infarction

The infarct size 14 days post‐MI increased significantly compared with the sham group (Figure 5). Treatment with HBO‐induced exosomes and overexpression of antagomir‐92a significantly decreased infarct size compared with the AMI group. AMI significantly increased left ventricular end‐diastolic and end‐systolic dimension and decreased fraction shortening compared with the sham control. Treatment with HBO‐induced exosomes and overexpression of antagomir‐92a significantly decreased left ventricular end‐diastolic and end‐systolic dimension and reversed the fraction shortening compared with MI alone at 14 days (Table 1). Transfection of MALAT1 LNA GapmeR did not reduce left ventricular end‐systolic and end‐diastolic dimension or improve fraction shortening.

**Table 1:** Haemodynamic and echocardiographic parameters

	Sham	AMI 1D	AMI 7D	AMI 14D	AMI 14D + non‐HBO‐exosome	AMI 14D + HBO‐exosome	AMI 14D + MALAT1 LNA GapmeR + HBO‐exosome	AMI 14D + scrambleLNA GapmeR + HBO‐exosome	AMI 14D + AntagomiR miR‐92a
N	5	5	5	5	5	5	5	5	5
Body weight, g	321 ± 26	322 ± 31	302 ± 36	276 ± 38	268 ± 43	304 ± 34	282 ± 49	297 ± 32	310 ± 33
Heart weight, mg	802 ± 21	821 ± 35	855 ± 47	859 ± 46	859 ± 46	831 ± 41	865 ± 52	829 ± 44	811 ± 49
Heart weight/body weight, mg/g	2.4 ± 0.6	2.5 ± 0.4	2.9 ± 0.9	3.1 ± 0.5	3.1 ± 0.8	2.6 ± 0.8	3.0 ± 0.7	2.6 ± 0.7	2.7 ± 0.4
Heart rate, min	317 ± 21	437 ± 55^+^	404 ± 48^+^	385 ± 49^+^	385 ± 49	341 ± 33	392 ± 26	350 ± 40	349 ± 28
MAP, mm Hg	115 ± 12	79 ± 9^+^	91 ± 6^+^	95 ± 5^+^	93 ± 9	103 ± 6	92 ± 3	105 ± 7	102 ± 7
LVEDD, mm	6.3 ± 0.4	6.9 ± 0.6	7.0 ± 0.4	7.4 ± 0.3^+^	7.3 ± 0.5	6.5 ± 0.3*	7.5 ± 0.65	6.7 ± 0.84*	6.4 ± 0.95*
LVESD, mm	2.7 ± 0.5	4.2 ± 0.3	4.4 ± 0.5	4.7 ± 0.4^+^	4.7 ± 0.5	3.3 ± 0.7*	4.7 ± 0.6	3.3 ± 0.4*	3.3 ± 0.4*
FS, %	57 ± 5	40 ± 6	36 ± 4^+^	36 ± 4^+^	35 ± 7	49 ± 7*	37 ± 6	50 ± 5*	49 ± 76*

Abbreviations: FS, fraction shortening; LVEDD, left ventricular end‐diastolic dimension; LVESD, left ventricular end‐systolic dimension; MAP, mean arterial pressure.

^+^ *P* < .05 vs sham.

* *P* < .05 vs AMI 14D.

### 3.4 MiR‐92a decreases KLF2 luciferase activity in cardiac myocytes under hypoxic conditions

**Figure 6:**
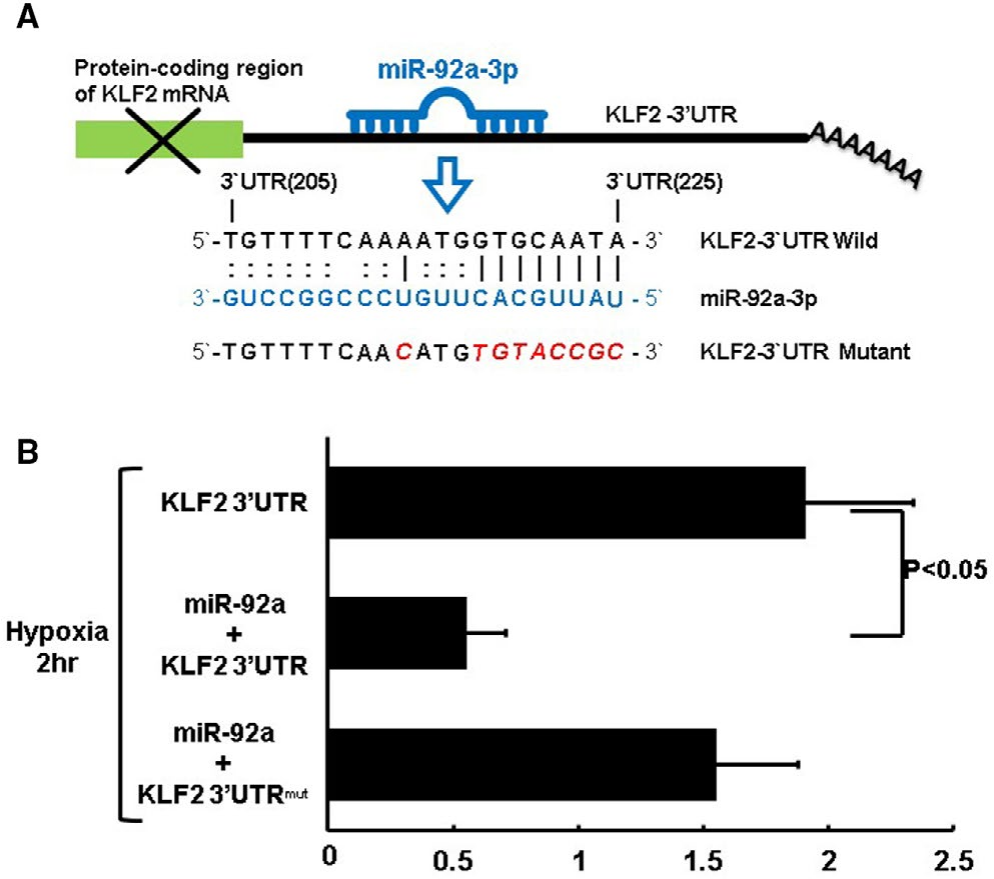
Effect of miR‐92a on KLF2 3′UTR luciferase activity in cardiac myocytes under hypoxic conditions. A, Sequence of the conserved miR‐92a‐binding site in the KLF2 3′UTR (nucleotides 205‐225). MiR‐92a inhibits translation and/or negatively regulates KLF2 mRNA stability by binding to the KLF2 3′UTR that cause suppression of KLF2 expression. B, KLF2 3′UTR luciferase activity under hypoxic conditions with or without miR‐92a expression (n = 4 per group)

To examine whether miR‐92a‐mediated reduction in KLF2 expression was regulated at the transcriptional level, luciferase activity of KLF2 was measured. The KLF2 3’UTR was found to contain a conserved miR‐92a‐3p‐binding site (nucleotide 205‐225; Figure 6A) using Clustal W method with the MegAlign software (DANSTAR, Madison, WI, USA). As shown in Figure 6B, miR‐92a overexpression significantly decreased KLF2 luciferase activity in cardiac myocytes under hypoxic treatment for 2 hours when miR‐92a bound to normal 3’UTR of KLF2 (Figure 6). The overexpression of miR‐92a in mutant KLF2 3’UTR did not affect the luciferase activity, indicating that KLF2 was the target gene of miR‐92a.

### 3.5 Effect of hypoxia on MALAT1, miR‐92a and KLF2 expression in cardiac myocytes

To mimic hypoxia induced by the ligation of the LAD artery, cardiac myocyte cultures were subjected to in vitro hypoxia with 2.5% oxygen. Hypoxia (with 2.5% oxygen) and HBO (at 2.5 ATA) for 2 hours significantly increased MALAT1 expression in the cardiac myocytes (Figure S3). HBO increased MALAT1 expression more than hypoxia. The hypoxia time course experiment revealed that MALAT1 expression in the cardiac myocytes gradually increased under hypoxia between 1 and 2 hours, then gradually returned to the baseline level (Figure S4A). In contrast to MALAT1 expression, miR‐92a expression initially decreased between 1 and 2 hours, and then gradually increased from 4 to 8 hours (Figure S4B). Hypoxia significantly increased KLF2 mRNA expression in the cardiac myocytes at 2 and 4 hours of hypoxia (Figure S4C). Treatment of cardiac myocytes, under hypoxia for 8 hours, with HBO‐induced exosomes significantly increased KLF2 mRNA expression while overexpression of miR‐92a in hypoxic cardiac myocytes significantly decreased KLF2 mRNA expression compared with the control group (Figure S5).

## 4 DISCUSSION

In our rat model of AMI, we found that the expression of MALAT1 increased, while that of miR92a decreased at the very early stages. Conversely, the expression of MALAT1 decreased and that of miR92a increased at the later stage. Our in vitro and in vivo studies demonstrated that HBO enhanced MALAT1 expression in the cardiac myocytes. Treatment with HBO or HBO‐induced exosomes significantly increased MALAT1 expression, whereas treatment with HBO‐induced exosomes significantly attenuated miR‐92 expression. Our AMI model showed increased left ventricular size and decreased fraction shortening, indicating a myocardial remodelling process. Treatment with HBO‐induced exosomes or miR‐92 antagonism significantly decreased the infarct size compared with AMI alone. Treatment with HBO‐induced exosomes or miR‐92 antagonism also reduced the left ventricular size and improved fraction shortening. LncRNAs not only modulate miR expression, but also function as miR targets, compete as decoys or sponge endogenous RNA to prevent the inhibition of mRNA targets.^20^, ^21^ We demonstrate that miR‐92a is a target of MALAT1 in human coronary artery endothelial cells.^22^ The present study further confirms that HBO increases MALAT1 expression and that the increased MALAT1 suppresses miR‐92a expression in the rat AMI model. MiR‐92a has been reported to play an important role in angiogenesis because ectopic expression of miR‐92a in endothelial cells inhibited angiogenesis, while the inhibition of endogenous miR‐92a increased angiogenesis.^23^, ^24^ The overexpression of miR‐92a significantly decreased CD31 expression in rats without AMI while treatment with antagomir‐92a significantly increased CD31 expression compared with AMI alone. CD31, an endothelial cell adhesion molecule and a marker for endothelial cells, is a crucial component of angiogenesis. The increased MALAT1 and decreased miR‐92a expression in the rat model of AMI may explain the improvement in angiogenesis and reduction of infarct size following HBO therapy. We have previously demonstrated that HBO increases exosomal MALAT1 and vascular endothelial growth factor receptor 2 to facilitate angiogenesis in human coronary artery endothelial cells.^22^ The inhibition of miR‐92a by antagomir‐92a has been reported as a therapeutic strategy for promoting skin repair in healing‐impaired diabetic mice.^25^ Exosomes are secreted by cardiac and vascular cells in culture.^26^ Exosomes were found to mediate communication between endothelial cells and smooth muscle cells, and fibroblasts and cardiac myocytes.^27^, ^28^ Boosting MALAT1‐containing exosomes may be used to augment angiogenesis post‐MI. Exosomes possess desirable properties such as stability, biocompatibility, biological barrier permeability, low toxicity and low immunogenicity, which make them an attractive vehicle for therapeutic delivery. MiR15a has been shown to inhibit angiogenesis.^29^, ^30^ We searched and found that rat MALAT1 has a matching site for miR‐15a‐5p and it is located from 4160 to 4178 bp of rat MALAT1 gene loci. Our study found that HBO‐induced exosomes could significantly attenuate the miR15a expression induced by hypoxia in cardiac myocytes (Figure S6). MALAT1 LNA GapmeR significantly reversed miR15a expression that was attenuated by HBO‐induced exosomes. Scrambled LNA GapmeR of MALAT1 did not reverse miR15a expression.

The role of MALAT1 in AMI is controversial. One study reported that the expression of MALAT1 increased in myocardial tissue post‐MI in mice and the increased MALAT1 protected cardiac myocytes from apoptosis.^31^ However, four other studies reported that the expression of MALAT1 increased post‐MI, but the increased MALAT1 promoted apoptosis of cardiac myocytes in mice.^32^, ^33^, ^34^, ^35^ MALAT1 promoted cardiac myocytes apoptosis through different miR targets.^32^, ^33^, ^34^, ^35^ In human studies, the blood level of MALAT1 was higher in patients with AMI than in healthy controls.^36^, ^37^ The reason of the discrepancy among different studies is not known. Different animal studies may partially explain the discrepancy. As most lncRNAs are not conserved among species,^38^ it is not surprising that MALAT1 expression decreases in myocardial tissue post‐MI in rats. The biological function of MALAT1 in enhancing neovascularization is the same among species. Silencing of MALAT1 reduced capillary growth in a mouse model of hindlimb ischaemia^13^ and rat model of diabetic retinopathy.^39^ The biological function of MALAT1 in enhancing angiogenesis is consistent with our findings.

KLF2 has been reported to be a target gene of miR‐92a^38^ and plays a critical role in neovascularization.^40^, ^41^ In this study, we observed that miR‐92a has a binding site in the 3’UTR of KLF2 and that it suppresses KLF2 luciferase activity by binding to the 3’UTR. KLF2 and CD31 expression decreased at 14 days post‐MI while HBO‐induced exosomes increased KLF2 and CD31 expression in left ventricular myocardium. HBO‐induced exosomes significantly increased KLF2 and CD31 expression post‐MI. Transfection with MALAT1 siRNA significantly decreased KLF2 and CD31 expression in HBO‐induced exosomes treatment group, whereas transfection with scrambled siRNA did not. Treatment with antagomir 92a significantly increased KLF2 and CD31 expression in left ventricular myocardium post‐MI. Transfection with KLF2 siRNA significantly decreased CD31 protein expression in HBO‐induced exosomes treatment group These findings indicate that HBO‐induced exosomes up‐regulate MALAT1 to suppress miR‐92a and counteract the inhibitory effect of miR‐92a on KLF2 expression in cardiac myocytes. Targeting MALAT1 through HBO may improve the clinical outcome in patients following AMI in addition to the current clinical benefits of β‐adrenergic receptor antagonists, angiotensin‐converting enzyme or receptor blockers, and aldosterone antagonists.

**Figure 7:**
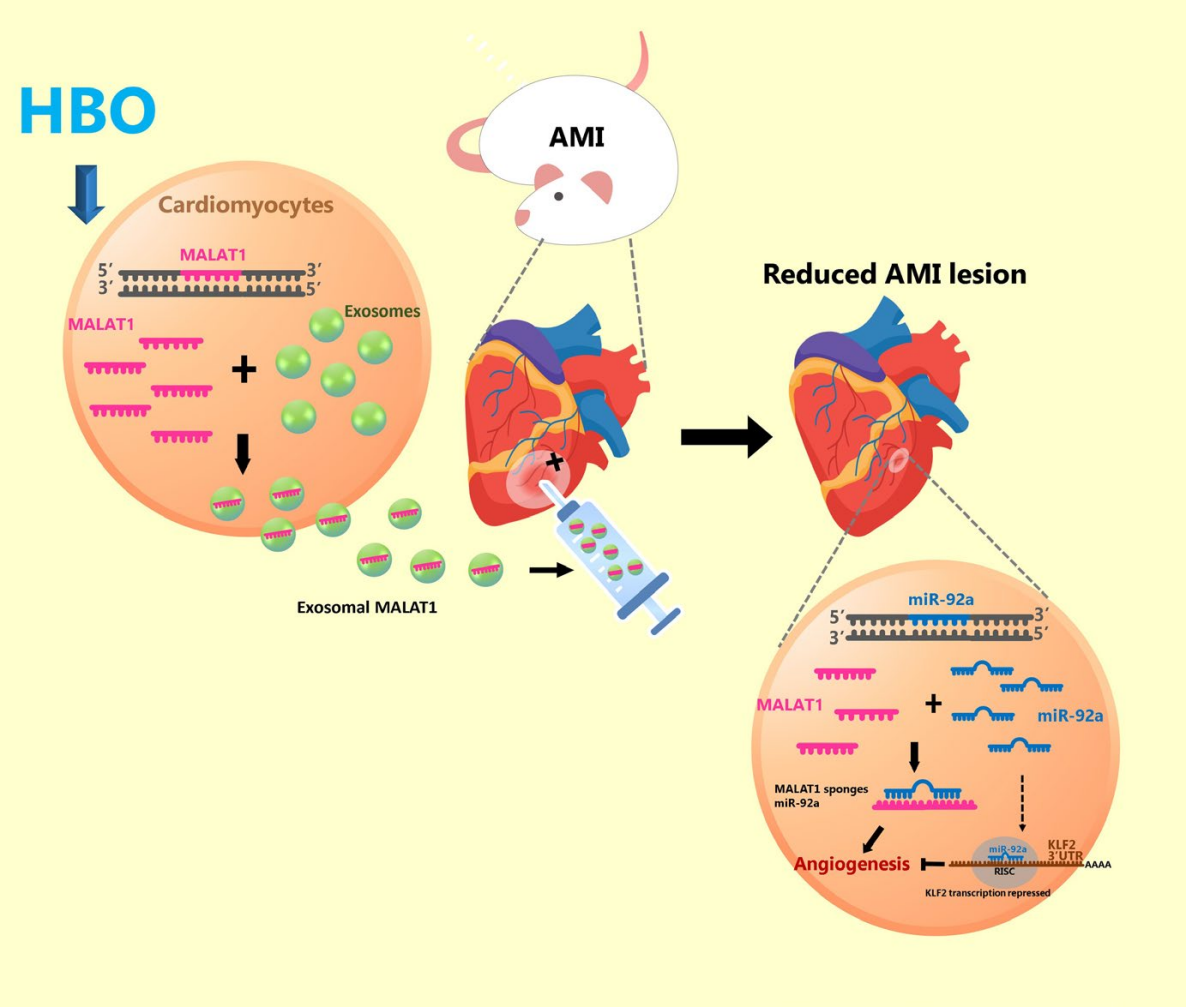
Proposed pathway of the enhancement of angiogenesis mediated by HBO‐induced cardiac myocyte‐derived exosomes through the up‐regulation of MALAT1, consequent suppression of miR‐92a expression and release of the inhibitory effect of miR‐92a on KLF2 expression in cardiac myocytes. This pathway illustrates the critical role of MALAT1 post‐myocardial infarction. MALAT1 can therefore serve as a valuable therapeutic tool for angiogenesis through HBO

In conclusion, HBO‐induced exosomes from cardiac myocytes up‐regulate MALAT1 to suppress miR‐92a expression and counteract the inhibitory effect of miR‐92a on KLF2 and CD31 expression in left ventricular myocardium post‐MI to enhance neovascularization (Figure 7). HBO‐induced exosomes containing MALAT1 may serve as a valuable therapeutic tool for neovascularization post‐MI.

## CONFLICTS OF INTEREST

The authors confirm that there are no conflicts of interest.

## AUTHOR CONTRIBUTION

**Kou‐Gi Shyu:** Funding acquisition (lead); Writing‐original draft (lead). **Bao‐Wei Wang:** Conceptualization (equal); Project administration (equal). **Wei‐Jen Fang:** Data curation (equal); Formal analysis (equal). **Chun‐Ming Pan:** Methodology (equal); Validation (equal). **Chiu‐Mei Lin:** Validation (equal); Writing‐review & editing (equal).

## Supporting information

Fig S1

Fig S2

Fig S3

Fig S4

Fig S5

Fig S6

## Data Availability

The data that support the findings of this study are available from the corresponding author upon reasonable request.
