# Supplementary figures and images for: Hyperbaric oxygen‐induced long non‐coding RNA MALAT1 exosomes suppress MicroRNA‐92a expression in a rat model of acute myocardial infarction
Source: J Cell Mol Med. 2020 Sep 16;24(22):12945–54. doi: 10.1111/jcmm.15889 (PMC7701534; doi:10.1111/jcmm.15889)

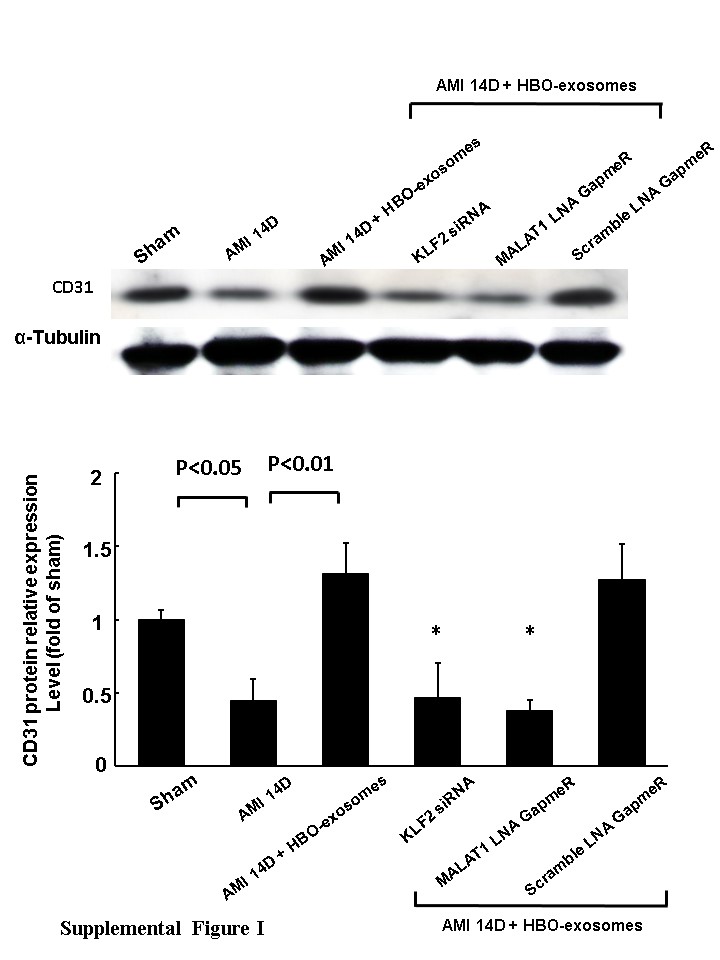

Supplement: Supplementary file 1 — Fig S1 [file JCMM-24-12945-s001.jpg]

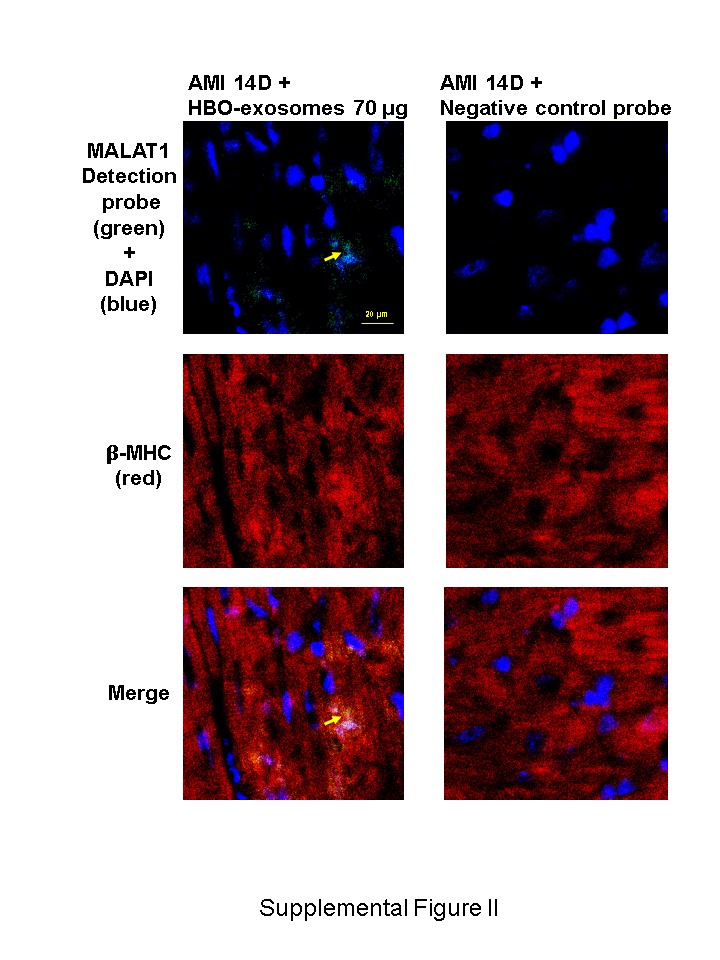

Supplement: Supplementary file 2 — Fig S2 [file JCMM-24-12945-s002.jpg]

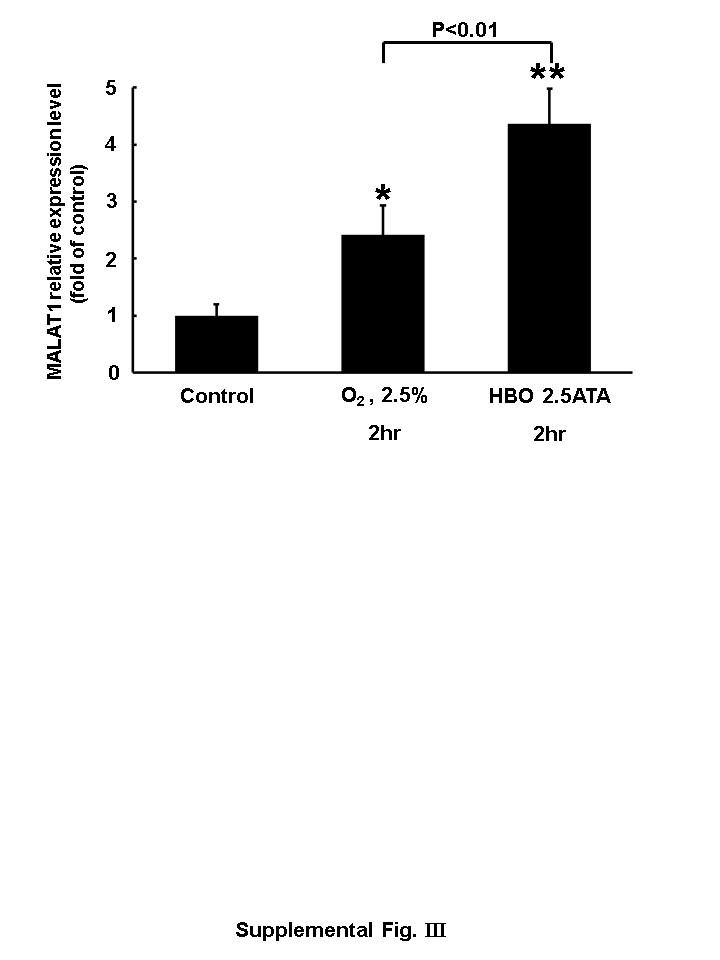

Supplement: Supplementary file 3 — Fig S3 [file JCMM-24-12945-s003.jpg]

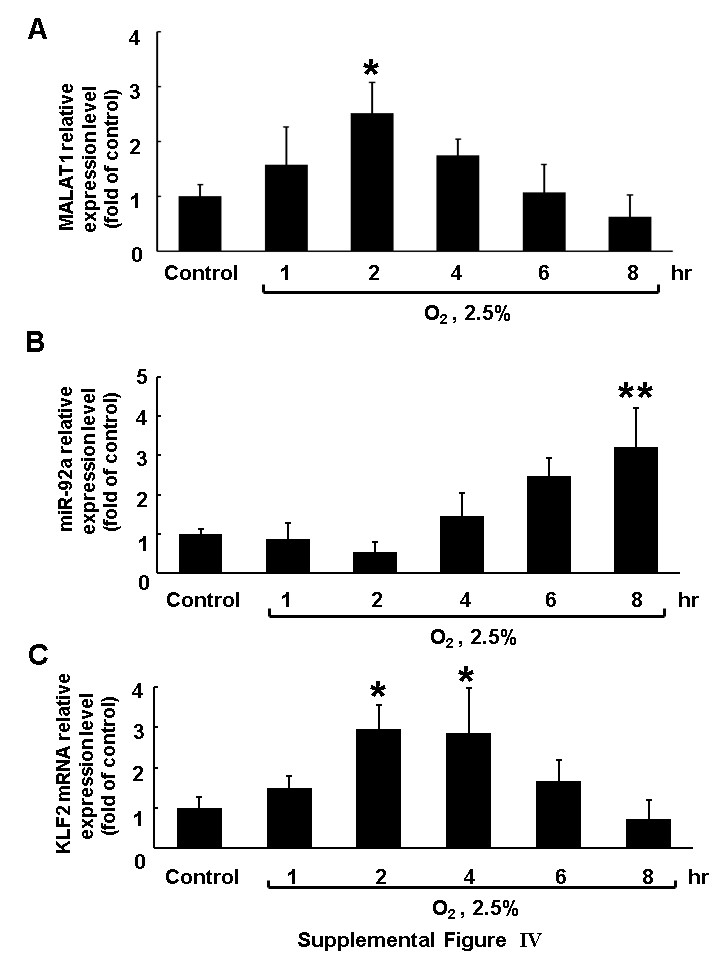

Supplement: Supplementary file 4 — Fig S4 [file JCMM-24-12945-s004.jpg]

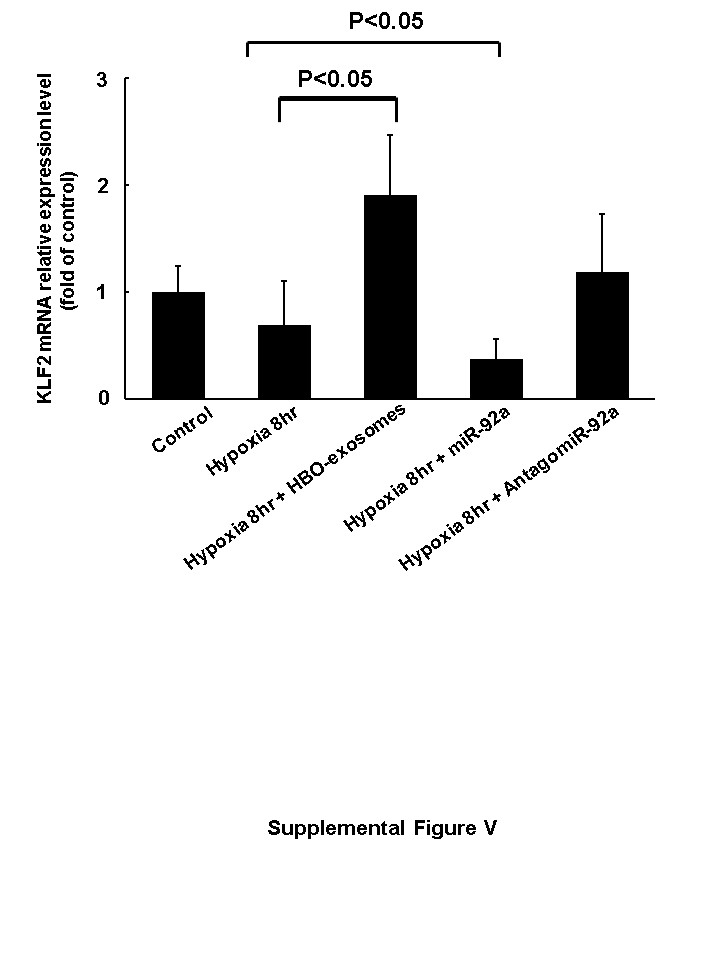

Supplement: Supplementary file 5 — Fig S5 [file JCMM-24-12945-s005.jpg]

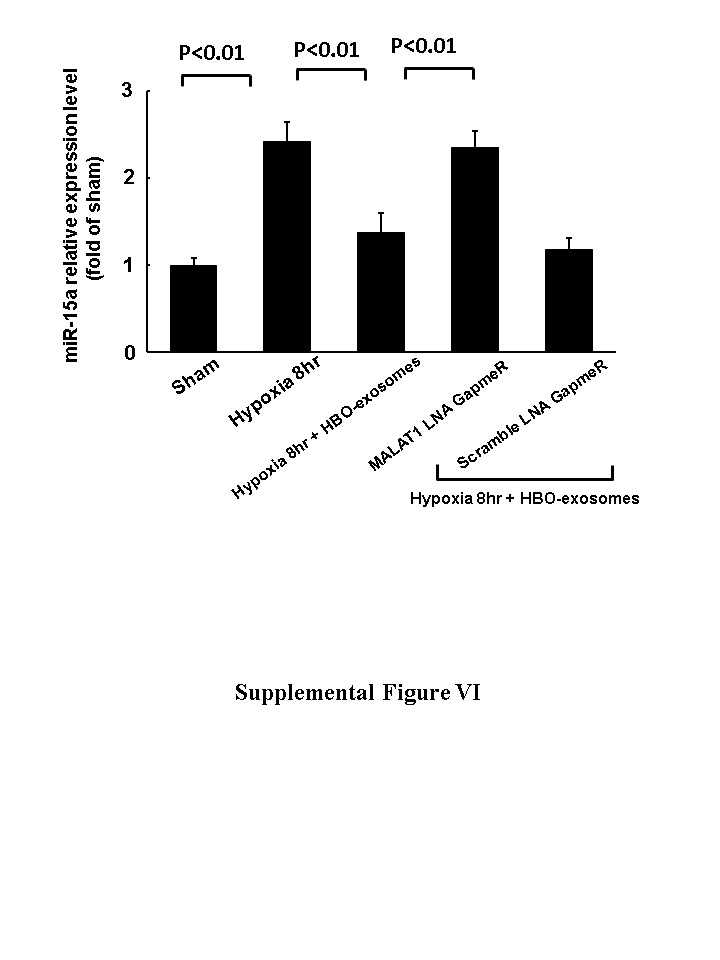

Supplement: Supplementary file 6 — Fig S6 [file JCMM-24-12945-s006.jpg]
